# Evolution of catastrophic health expenditure in a high income country: incidence versus inequalities

**DOI:** 10.1186/s12939-019-1044-9

**Published:** 2019-09-18

**Authors:** Carlota Quintal

**Affiliations:** FEUC, CeBER, CEISUC, Faculty of Economics, University of Coimbra, Av. Dias da Silva, 165 |, 3004-512 Coimbra, Portugal

**Keywords:** Catastrophic health expenditure, Financial protection, Inequality, Portugal

## Abstract

**Background:**

Catastrophic health expenditure (CHE) is well established as an indicator of financial protection on which there is extensive literature. However, most works analyse mainly low to middle income countries and do not address the different distributional dimensions of CHE. We argue that, besides incidence, the latter are crucial to better grasp the scope and nature of financial protection problems. Our objectives are therefore to analyse the evolution of CHE in a high income country, considering both its incidence and distribution.

**Methods:**

Data are taken from the last three waves of the Portuguese Household Budget Survey conducted in 2005/2006, 2010/2011 and 2015/2016. To identify CHE, the approach adopted is capacity to pay/normative food spending, at the 40% threshold. To analyse distribution, concentration curves and indices (CI) are used and adjusted odds ratios are calculated.

**Results:**

The incidence of CHE was 2.57, 1.79 and 0.46%, in 2005, 2010 and 2015, respectively. CHE became highly concentrated among the poorest (the respective CI evolved from − 0.390 in 2005 to − 0.758 in 2015) and among families with elderly people (the absolute CI evolved from 0.520 in 2005 to 0.740 in 2015). Absolute CI in geographical context also increased over time (0.354 in 2015, 0.019 in 2005). Medicines represented by far the largest share of catastrophic payments, although, in this case concentration decreased (the median share of medicines diminished from 93 to 43% over the period analysed). Contrarily, the weight of expenses incurred with consultation fees has been growing (even for General Practitioners, despite the NHS coverage of primary care).

**Conclusions:**

The incidence of CHE and inequality in its distribution might progress in the same direction or not, but most importantly policy makers should pay attention to the distributional dimensions of CHE as these might provide useful insight to target households at risk. Greater concentration of CHE can actually be regarded as an opportunity for policy making, because interventions to tackle CHE become more confined. Monitoring the distribution of payments across services can also contribute to early detection of emerging (and even, unexpected) drivers of catastrophic payments.

## Background

The financial protection of families, when using needed healthcare, has been long recognised as a core dimension of health system evaluation [1, 2], becoming part of the 17 Sustainable Development Goals promoted by the United Nations in 2015 [3]. In this context, catastrophic health expenditure (CHE) is now an established indicator of the financial protection dimension. It is defined as out-of-pocket payments (OOP) that exceed a predefined percentage or threshold of a household’s resources [4, 5].

Since the seminal work by Xu et al. [6], a large body of literature on the incidence of CHE has emerged and developed. However, in a systematic review of literature [7] several gaps were identified, namely the scarcity of up-to-date analysis and a bias of the literature towards middle-income countries. Few studies analysed trends over time and not many developed equity analyses (and most equity analyses correspond to the calculation of CHE by expenditure or income quintiles). Hence, we aim to contribute to the literature by analysing the evolution of CHE in a high income country over a decade (from 2005 to 2015). By following exactly the same methodological steps in the analysis of the three surveys, this study provides assurance of the comparability of results. Moreover, our aim is to focus not only on incidence figures but also on distributional aspects which have been less explored. In the latter case, we consider income-related inequalities in CHE but we also analyse the distribution of CHE across geographic areas and across family types. We further look at the distribution of the health expenditure of families incurring CHE across types of healthcare.

Previous evidence suggests considerable country variation of the incidence of CHE by income group. Specifically, Xu et al. [8] obtained a median incidence of 1.47%, significantly below the average of 2.3% (this difference being explained by the fact that a few countries had rates over 4%, while most were below 2%). Curiously, Portugal was one of the four examples given by the authors regarding countries that, in spite of being members of the OECD, had rates in excess of 0.5%. This might be partly explained by the high prevalence of direct payments in Portugal. In fact, it is among the OECD countries with the highest shares of OOP in total health expenditure. In 2005 this share was 22% [9], amounting to 28% in 2015 [10]. The OECD average was 20% in both years. The country’s National Health Service (NHS) is universal, comprehensive and almost free at point of delivery (according to the Portuguese Constitution, Article 64). Although no services are explicitly excluded from NHS coverage, there are shortcomings in provision which in turn explain the share of direct payments in total health expenditure. The NHS predominantly provides primary care and specialized hospital care but it does not cover dental care (which is neither provided nor funded by the NHS). Most dental care is paid by OOP, as are many specialist consultations in private ambulatory care [11]. The private sector still plays a relevant role in healthcare provision in Portugal which might be explained by both NHS shortages (with long waiting times) and a tradition, from before the creation of the NHS, of direct access to physicians’ private practices [11]. Regarding pharmaceutical expenditure, there are cost-sharing schemes [11]. However, the public share of expenditure on retail pharmaceuticals decreased from 59% in 2005 [9] to 55% in 2015 [10].

During the period analysed in this study, Portugal was hit by the 2008 economic crisis. The effects of the economic recession were aggravated by the public debt crisis. Following the financial rescue plan of the country, several reforms were implemented in the health sector in order to reduce costs and increase efficiency [11–13]. From the standpoint of CHE, the impact of the crisis and of these changes in the health sector is not straightforward. User charges were increased but exemptions were also extended [12, 14, 15]. Tighter rules for cost-sharing in pharmaceuticals were put in place, but average medicine prices decreased [15]. Catastrophic health expenditure is also affected by household resources. Therefore, the economic crisis possibly affected catastrophic spending by impacting family income. In 2015, 19.0% of the Portuguese population was living below the poverty line, defined as 60% of the median income. This proportion of the population had been growing since 2010, when it stood at 18.0% [11]. On the other hand, during times of financial hardship, families, aware of their arrear difficulties, might restrain their consumption of healthcare services and goods and, consequently, they reduce their OOP. In this context, our work aims to analyse the evolution of CHE by addressing both incidence and distribution, based on data from before, during and after the economic and public debt crisis.

## Methods

### Data

Data are taken from the 2005/2006, 2010/2011 and 2015/2016 waves of the Portuguese Household Budget Survey (PHBS). This survey contains data from a representative stratified clustered sample of households living in non-collective dwellings across the country. It is carried out by Statistics Portugal. The series of surveys on household budgets initiated in Portugal in 1967/1968, and the PHBS began in 2005/2006. Data have been collected every five years. Besides information on expenditure, the PHBS also collects demographic and income data. In 2010/2011, the nomenclature from the Classification of Individual Consumption by Purpose was adopted as well as the electronic recording during the collection of daily consumption of goods and services.

The data used in this study were collected between 10 October 2005 and 8 October 2006 [16], between 1 March 2010 and 27 February 2011 [17] and between 16 March 2015 and 13 March 2016 [18]. In the text hereafter and to simplify, we will refer to the above as 2005, 2010 and 2015, respectively. The three samples used in this study contain data regarding 10,403, 9489 and 11,398 households, in chronological order of the surveys.

The variables extracted from the databases concern: total expenditure, expenditure for food, out-of-pocket healthcare payments (total and for different types of healthcare considered separately: medicines and other pharmaceutical products; medical consultations – in the 2015 survey, this information is broken down further by general practitioners (GP) and specialist visits; dental care; diagnostic tests; paramedic services; hospital services), monetary income, type of household (one non-elder adult, one elder adult, two or more non-elder adults, two or more adults with at least one elder individual and no children, one adult with children, two or more adults with one child, two or more adults with two or more children), household size, sex and level of education (basic, secondary, superior) of head of household, and region of residence (rural versus urban and NUTs II: North, Centre, Lisbon Metropolitan Area, Alentejo, Algarve, Azores and Madeira).

### Empirical analysis

To identify households with CHE, we adopt the methodology proposed by WHO researchers [6, 19] and followed by several authors (such as Yardim et al. [20], Ozgen et al. [21], Masood et al. [22], and more recently Zawada et al. [23] and Bernabé et al. [24], to name a few). In this case, a household is said to have incurred catastrophic expenditure if its OOP are equal to, or higher than, 40% of its capacity to pay. Capacity to pay corresponds to a household’s non-subsistence spending. Subsistence spending is the amount each household is expected to spend on food taking into account its equivalent size and the amount spent by the household on the sample median food share of total expenditure. The detailed steps of the calculation of CHE can be found in Xu [19].

Before analysing inequalities in the distribution of CHE and in order to get an idea of the wider scenarios behind catastrophic payments over the years, we start by looking at the distribution of households across different bands of shares of OOP in total capacity to pay as well as the distribution of these shares across households ranked by capacity to pay. In the latter case, we present the concentration curves for the three periods considered in our study. The concentration curve plots the cumulative percentage of, in this case, shares of OOP (y-axis) against the cumulative percentage of the population (in our case, households), ranked by living standards (in our case, proxied by capacity to pay), beginning with the poorest, and ending with the richest (x-axis) [25].

To analyse income-related inequalities in the distribution of CHE, we present also concentration curves for the three periods considered in our study. Here, we have in the vertical axis the cumulative percentage of CHE, and the horizontal axis there shows the cumulative percentage of households ranked by capacity to pay.

To quantify the degree to which the distribution of CHE departs from proportionality we resort to the calculation of the concentration index (CI). CI equals twice the area between the concentration curve and the line of equality (the 45° line running from the bottom-left corner to the top-right). Where there is no inequality, the CI is zero; a negative (positive) CI indicates a disproportionate concentration of the given variable (in our case, CHE) among the worse-off (well-off) [25]. In the case of the distribution of CHE across regions and family types, we rank groups starting with highest CHE, but there is no such interpretation as concentration among the worse-off (better-off). Thus, we report the absolute value of the CI as what matters here is the magnitude of inequality.

In the case of the distribution of CHE according to households’ capacity to pay, we estimate the CI following the convenient regression approach [25, 26]. In the case of the distribution of CHE according to geographic areas and types of households, we calculate the concentration index following the suggestion for the case of grouped data [25].

Given that CHE is a binary variable, we present the results also for the corrected index suggested by Wagstaff [27, 28]: if CI is the standard concentration index, the corrected concentration index (CCI) is equal to $\frac{CI}{1-\tau }$, where *τ* is the mean of the variable being analysed.

To better understand where concentration occurs in the case of the distribution of CHE across regions and family types, we adopt multiple logistic regression analysis. The results of the regression analysis are reported as adjusted odds ratios (AOR); the ratio of the odds of an event (CHE) occurring in one group compared to the odds of that event occurring in another group (reference category), controlling for other explanatory variables. In the geographic analysis we report AOR for all regions (Madeira is the reference) and in the case of family type we report AOR for households with at least one elder. Remainder explanatory variables included are: household size, presence of children, sex and level of education of head of household, living in rural (versus urban) area and family monetary income.

To analyse the distribution of catastrophic payments across services and goods, we calculate, for the subsample of families incurring CHE, the frequencies of shares of different kinds of expenditure in total healthcare expenditure (full list of services and goods provided in previous section). When the share of a given item is 1, this means that households presenting this share incur catastrophic expenditure by spending on this item alone.

Finally, with the objective of complementing the previous analysis, we assess the distribution of healthcare payments in relation to capacity to pay (now using whole samples), calculating the Kakwani index (or progressivity index) for total payments and then for medicines, consultations, dental care and exams. If healthcare payments are proportional to capacity to pay, the Kakwani index is zero; if payments are regressive (progressive) then the index is negative (positive) thus indicating a disproportionate concentration of payments among the poor (rich). The Kakwani index is also estimated with the convenient regression method [25, 26].

All the analyses are carried out in SPSS 25.0 and Excel (2013). Sample weights provided in the databases are used.

## Results

**Fig. 1:**
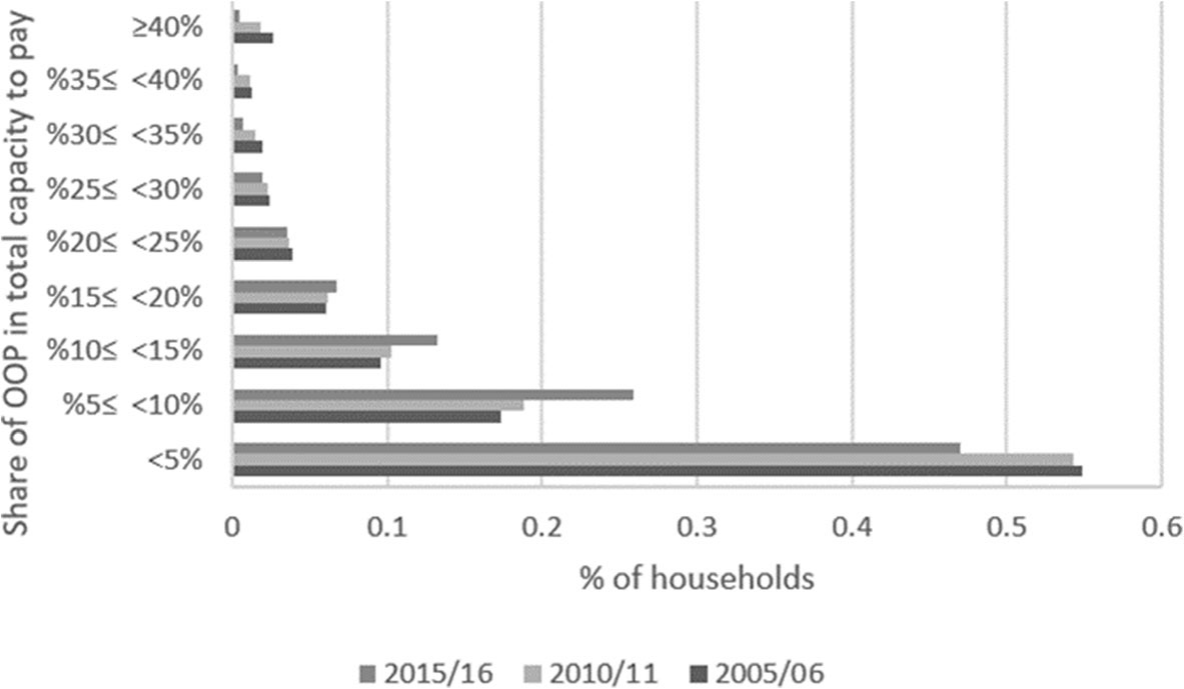
Distribution of households according to their shares of out-pocket-payments (OOP) in total capacity to pay

As shown in Fig. 1, most families spent up to 5% of their capacity to pay for healthcare. However, while in 2005 and 2010 this was the case for more than half of families, in 2015 less than 50% of households were in this group. In fact, we can observe that the percentage of families in the extreme bands (below 5% and above 30%) shrank from 2005 to 2015. On the other hand, the percentage of families spending on healthcare between 5 and 10% and between 10 and 20% of their capacity to pay increased.

**Fig. 2:**
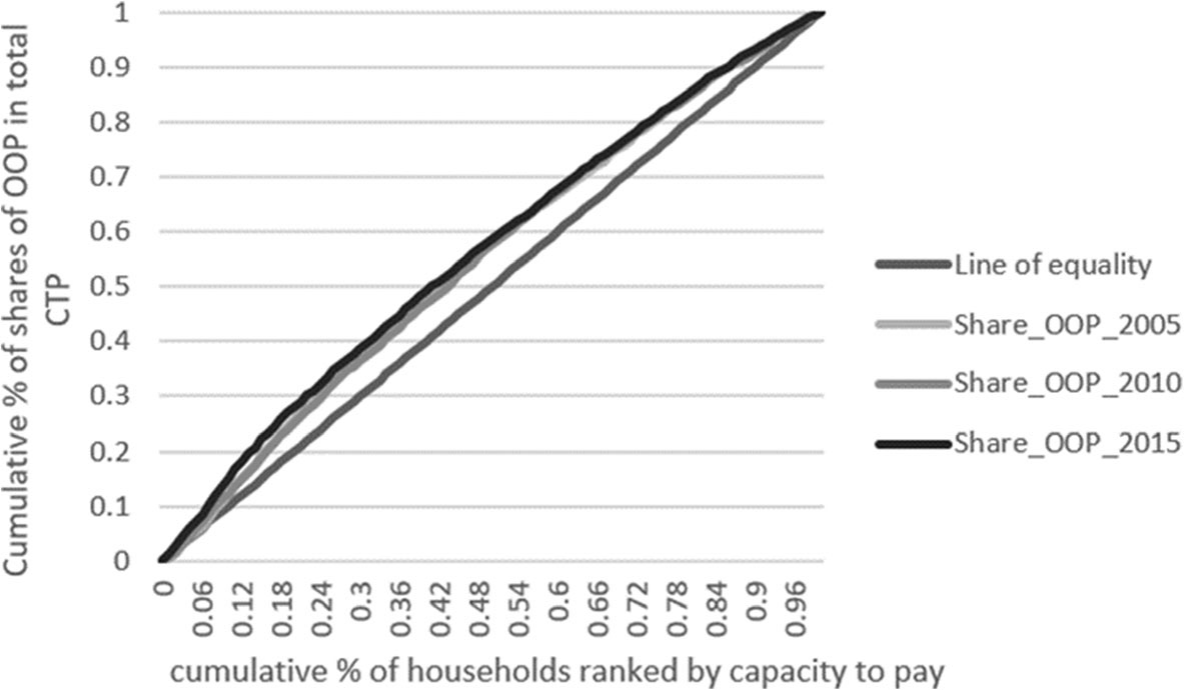
Concentration curves for shares of out-of-pocket expenditure (OOP) in total capacity to pay (CTP)

The distribution of the shares of OOP in total capacity to pay (Fig. 2) did not change much in a decade. The 5% poorest households accounted for 5, 6 and 7% of total shares of OOP, in 2005, 2010 and 2015, respectively. For the 10% poorest families, figures were the same in 2005 and 2010 (12% of total shares of OOP) but in 2015 the 10% poorest accounted for 15% of total OOP shares. However, as we consider successively higher shares of households, the differences between periods decrease (the 52% poorest families accounted for 60% of total shares of OOP in the three periods considered and the 20% richest bore 15% of total shares in all years).

**Fig. 3:**
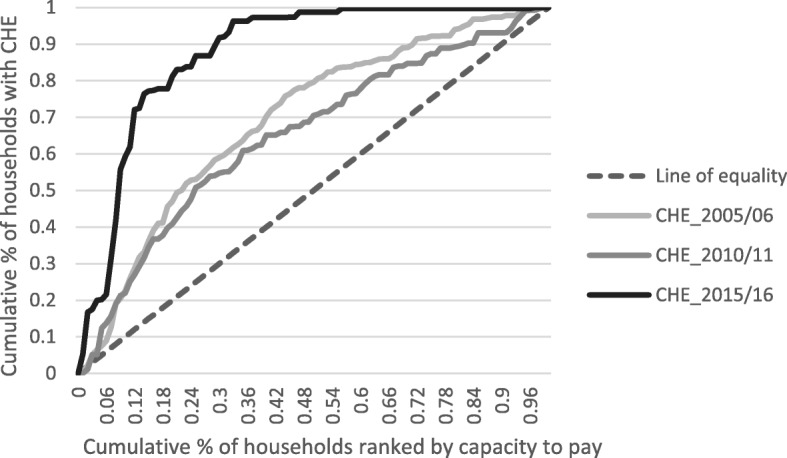
Concentration curves for catastrophic health expenditure (CHE)

The incidence of CHE in Portugal decreased over the period analysed in this study, from 2.57 to 0.46% (Table 1). The reduction in the incidence of CHE was particularly strong (over 70%) between 2010 and 2015. Regarding inequality in the distribution of CHE across households with different capacity to pay, results for the concentration index show that inequality firstly decreased (from 2005 to 2010) but then the concentration more than doubled. Figure 3 shows the same picture with the concentration curve for 2015 separating from the other two curves right in the bottom of the capacity to pay distribution. The 10% poorest households accounted for 22.6 and 21.9% of total cases of CHE, in 2005 and 2010, respectively. In 2015, the 10% poorest families accounted for 59% of total cases of CHE.

**Table 1:** Incidence of catastrophic health expenditure and its distribution: 2005–2015

	2005/06	2010/11	2015/16
CHE (SE)	2.57% (0.00008)	1.79% (0.00007)	0.46% (0.00003)
Distribution of CHE by:			
Capacity to pay			
CI (CCI)	−0.390^***^ (− 0.400)	− 0.299^***^ (− 0.294)	−0.758^***^ (− 0.762)
Regions NUT II			
|CI| (|CCI|)	0.019^***^ (0.019)	0.010^***^ (0.010)	0.354^***^ (0.356)
Household types			
|CI| (|CCI|)	0.520^***^ (0.534)	0.316^***^ (0.322)	0.740^***^ (0.743)

Notes: *CHE* catastrophic health expenditure, *CI* concentration index, *CCI* corrected concentration index, *|CI*| absolute value of concentration index, *|CCI|* absolute value of corrected concentration index; SE-standard error

*** *p* < 0.01

In terms of the distribution of CHE across regions and across household types, we found a similar pattern to that obtained previously: inequality decreased from 2005 to 2010 and then it significantly increased (Table 1).

**Fig. 4:**
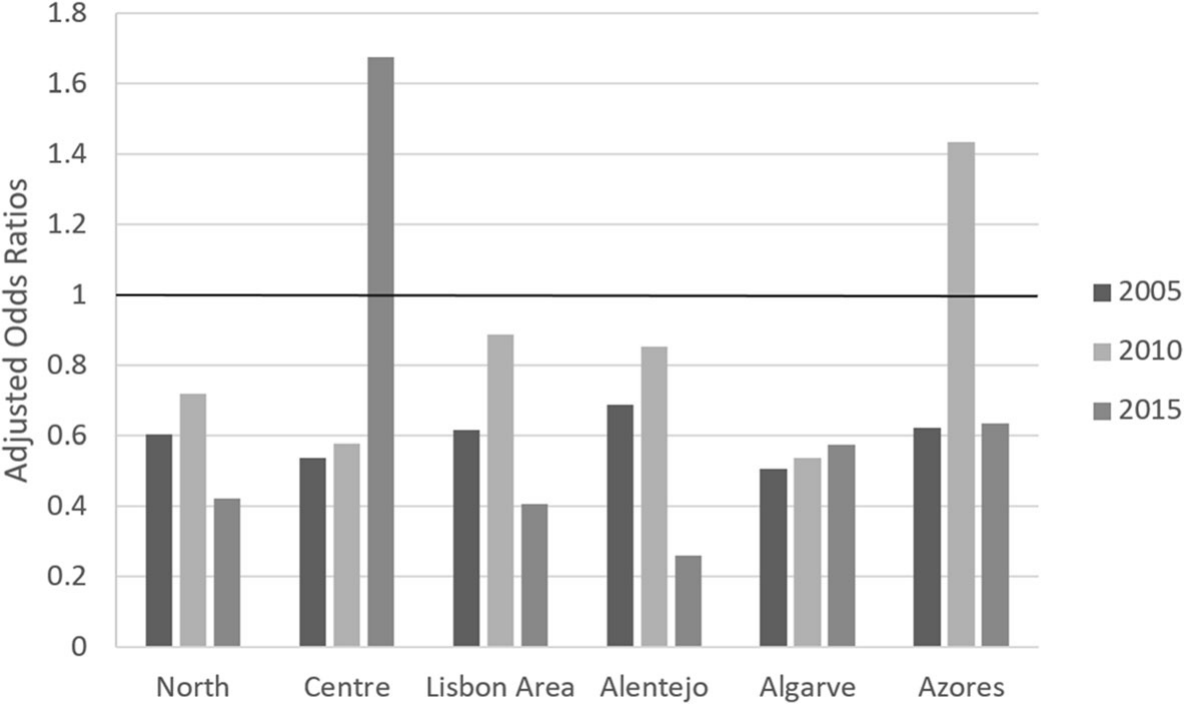
Risk of catastrophic expenditure across regions NUT II – Adjusted Odds Ratios: 2005/06; 2010/11 and 2015/16. *Notes:* Odds Ratios are adjusted for household annual monetary income, household dimension, level of education of head of household, the presence of at least one elder member in household, rural vs urban area. The reference category for Regions is Madeira. The line AOR = 1 corresponds to equal risk of CHE, compared to Madeira. All coefficients are statistically significant at 1% in 2005 and 2010; in 2015, only the coefficients for North (*p* < 0.1), Lisbon Area (p < 0.1) and Alentejo (*p* < 0.05) are statistically significant

Figure 4 provides some clues on the risk of CHE across regions. In 2005, the risk of CHE in all regions was lower than in Madeira (reference category) and it was the lowest in Algarve, followed by the Centre. In 2010, again Algarve and Centre were the regions with the lowest risks. Azores in turn presented the highest risk of CHE (higher than Madeira), showing a huge increase compared to 2005. In 2015, we got a different picture, with Alentejo showing by far the lowest risk and the Centre showing by far the highest risk; Azores returned to its 2005 level.

**Fig. 5:**
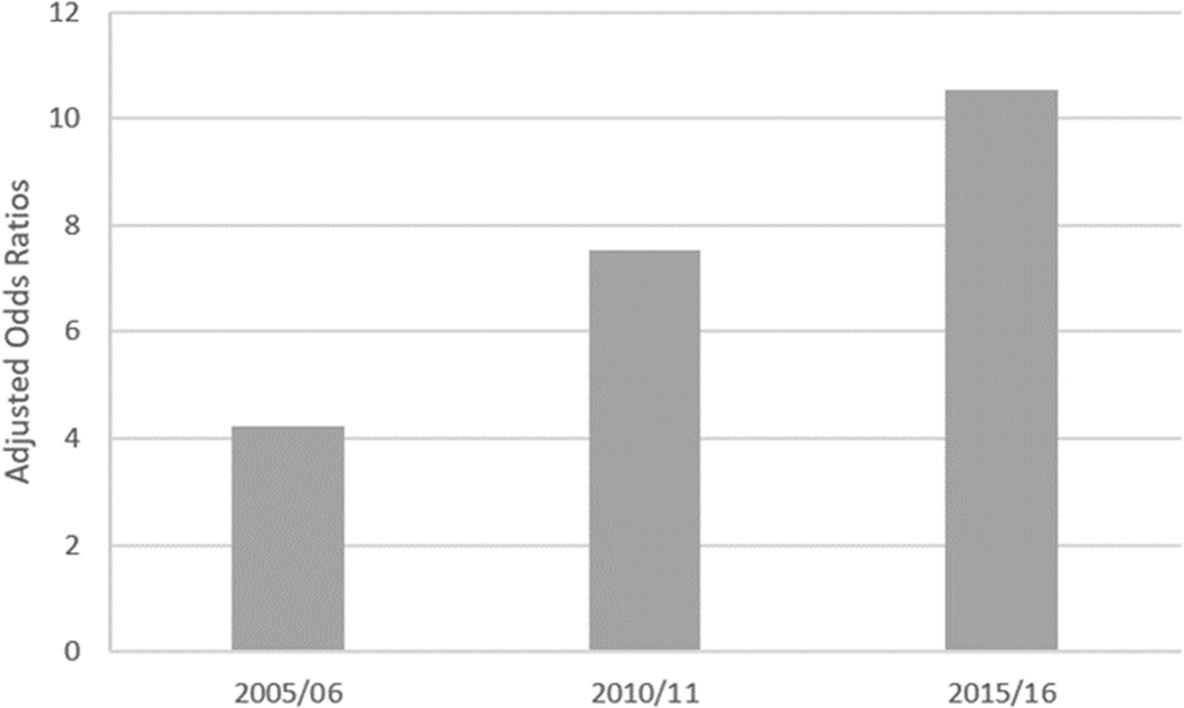
Risk of catastrophic health expenditure among households with at least one old member – Adjusted Odds Ratios: 2005/06, 2010/11 and 2015/16. *Notes:* Odds Ratios are adjusted for household annual monetary income, household dimension, the presence of children, sex and level of education of head of household, regions NUT II. All coefficients are statistically significant at 1%

Considering the association between the risk of CHE and household composition, Fig. 5 suggests that households with elderly members faced an accrued risk of CHE and that this risk increased from 2005 to 2015, when families with at least one elder were roughly 10 times more likely to incur CHE than families without elderly members.

In Table 1, differences between CI and CCI are quite small because mean values for CHE are low, hence, it is irrelevant to analyse one or the other indicator.

As seen in Table 2, the most relevant expense is undoubtedly medicines. First, very few families incurring CHE had null expenditure on medicines (in 2015/16, all families with CHE spent on medicines); second, medicines are by far the single item that had more households incur CHE. In 2005, 35.5% of families with CHE spent only on medicines. This percentage decreased to 33% in 2010 and then to 21.3% in 2015. Having families with CHE spending on a single item rarely occurred with other services. In 2015 it does not happen at all. In 2005, 2.3% of families with CHE spent only on hospital services and, in 2010, the same proportion spent only on dental care. The majority of families with CHE, in all surveys, did not spend money on paramedics, hospital services, exams and dentists. Regarding expenditure on medical consultations, the median share in total health expenditure is zero but the relevance of this item increased over time. In 2015, 25% of families incurring CHE devoted at least 24% of their total expenditure to medical consultations. In 2005, this figure was only 7%.

**Table 2:** Weights of different types of expenditure in total OOP among households incurring CHE: 2005–2015

	Minimum weight (% of HH)	1st quartile weight	Median weight	3rd quartile weight	Maximum weight (% of HH)
Medicines					
2005/06	0 (6%)	0.5	0.93	1	1 (35.5%)
2010/11	0 (6.4%)	0.3	0.76	1	1 (33%)
2015/16	0.04 (0.3%)	0.28	0.43	0.79	1 (21.3%)
Consultations					
2005/06	0 (64.5%)	0	0	0.07	0.83 (0.1%)
2010/11	0 (59.7%)	0	0	0.13	0.71 (1%)
2015/16	0 (49%)	0	0.08	0.24	0.83 (0.9%)
GPs	0 (64.4%)	0	0	0.13	0.70 (0.4%)
Spec.	0 (63.7%)	0	0	0.19	0.83 (0.9%)
Dentists					
2005/06	0 (84.7%)	0	0	0	1 (0.2%)
2010/11	0 (79.0%)	0	0	0	1 (2.3%)
2015/16	0 (74.6%)	0	0	0.09	0.88 (0.2%)
Exams					
2005/06	0 (82.9%)	0	0	0	0.91 (0.5%)
2010/11	0 (76.5%)	0	0	0	0.88 (0.5%)
2015/16	0 (76.7%)	0	0	0	0.6 (0.5%)
Paramedics					
2005/06	0 (93.9%)	0	0	0	0.91 (0.3%)
2010/11	0 (89.2%)	0	0	0	0.94 (0.8%)
2015/16	0 (86.0%)	0	0	0	0.59 (0.8%)
Hospital					
2005/06	0 (89.8%)	0	0	0	1 (2.1%)
2010/11	0 (94.5%)	0	0	0	0.86 (0.1%)
2015/16	0 (86.4%)	0	0	0	0.14 (0.5%)

Note 1: CHE – Catastrophic health expenditure; HH- Households; GPs- General Practitioners; Spec.- Specialists

Note 2: Zero (0) means that the service in question accounts for 0% of total health care expenditure (for example, 0 for ‘Consultations’ in the column ‘Median weight’ in 2005/06 means that at least 50% of families incurring CHE did not spend on medical consultations in this period); 0.13 for ‘GPs’ in the column ‘3rd quartile weight’ in 2015/16 means that in this period 75% of households with CHE used up to 13% of their health care payments to pay for GP consultations –it also means that the remainder 25% of households incurring CHE spent more than 13% of their total payments on GP consultations. The value of 1 means that the service in question represents 100% of out-of-pocket expenditures (for example, 1 for ‘Medicines’ in the column ‘Maximum weight’ means that some households incurring CHE spent on medicines alone – in 2005/06, this happened to 35.5% of households with CHE)

Table 3 shows that all payments (whole samples) were not proportional (all CI are statistically significant). Medicine payments were the most regressive and no relevant changes occurred in ten years. The Kakwani index for consultations was negative in 2005 and 2010, but it became positive and increased in 2015. By breaking down the latter, two different patterns are identifiable: payments for GP visits were regressive while payments for specialist visits were progressive.

**Table 3:** Progressivity indices for different types of expenditure: 2005–2015

	2005/06	2010/11	2015/16
Total OOP	−0.093^***^	−0.096^***^	−0.110^***^
Medicines	−0.250^***^	−0.266^***^	−0.241^***^
Consultations	−0.018^***^	−0.013^***^	0.023^***^
GPs	–	–	−0.061^***^
Specialists	–	–	0.060^***^
Dentists	0.076^***^	0.114^***^	0.035^***^
Exams	0.076^***^	0.013^***^	0.038^***^

OOP- out-of-pockets; GPs- General Practitioners

*** p < 0.01

## Discussion

When research on catastrophic health expenditure emerged in the literature [6, 8], the example of Portugal was highlighted as an OECD country with an unexpectedly high level of CHE. Countries whose share of OOP is between 20 and 30% of total health care expenditure are expected to have less than 1.5% of households with CHE [8], but in Portugal, there were more than 2.5%. Consequently, CHE in Portugal was too high even considering the relatively high level of OOP in the country. In fact, if we look at world figs. [29], the 2–3% band of CHE was populated by low and low-middle income countries, thus, Portugal was not supposed to be there. Our study analysed the evolution of CHE using posterior data, from 2005 to 2015, and after a decade Portugal finally achieved a level of CHE aligned with high income and developed countries.

Since we are dealing with a dichotomous analysis and because any threshold is arbitrary [4], a low incidence of CHE could mask financial problems of households just below the 40% threshold. However, as shown by our results, this was not the case given that the percentage of households spending 30% or more of their capacity to pay for healthcare significantly decreased in 2015 compared to both 2005 and 2010.

This remarkable progress in terms of the incidence of CHE occurred alongside a general increase in the inequality of its distribution, though with some differences in the two sub-periods. That is, from 2005 to 2010, there was a smoother decrease in the level of CHE and all types of inequality analysed in this study also decreased. Whereas from 2010 to 2015, there was a sharper reduction of CHE, but inequalities were very substantial.

Comparing our results with previous studies is a limited exercise as these distributional aspects of CHE have been less explored. Still, a recent study on Greece [30], based on the same methods adopted in the current paper, found that the incidence of CHE (for the 40% threshold of capacity to pay) increased from 1.03% in 2008 to 1.19% in 2015 although during that period it decreased to 0.49%, in 2012. Regarding inequalities, the CI obtained by authors fluctuated over time and it even changed from negative to positive (households were ranked by total expenditure). In 2008, the CI was − 0.233 and in 2015 it was 0.232. Although Greece experienced an economic and public debt crises, like Portugal, the observed behaviour of CHE in both countries is different. In 2015, Portugal had a significantly lower level of CHE than Greece, but a much higher level of inequality. Moreover, CHE in Portugal was disproportionately concentrated among the poorest while the opposite occurred in Greece. Zawada et al. [23] derived concentration curves for CHE for three countries. Their results are not fully comparable with ours as they considered in their inequality analysis a threshold (defining CHE) equal to 10% of total income. Still, the authors concluded that in Poland (year 2010) catastrophic OOP were concentrated among the poor, whereas in Denmark (year 2010) they were concentrated among the rich, while the disparities in Germany (year 2009) were variable, but close to the equality line. In Zawada et al. [23], the country with greater inequality, Poland, also had the largest incidence of CHE and the country with less inequality, Germany, also had the lowest incidence of CHE. Although in a completely different setting (low income country), a study for Tajikistan [31], using the same method as in our work, found that the incidence of CHE decreased from 31% in 2008 to 18.8% in 2011, while the level of inequality was relatively low, with the CI evolving from 0.008 in 2008 to − 0.072 in 2011.

In what concerns the distribution of CHE across types of households, our results suggest that CHE is concentrated on households with elderly, which is in line with previous evidence that found that the presence of elderly in households increased the risk of CHE in Portugal [32, 33] and elsewhere [34, 35]. Moreover, the risk of CHE faced by these households has expressively increased across the three survey periods. Combining this result with the confirmation that medicines represent the lion’s share when it comes to household CHE, the progress of the prevalence of chronic conditions might partly explain these findings. The prevalence of chronic conditions (and multimorbidity) in Portugal has grown [36]. For example, hypertension and diabetes, which are often associated with medication needs, had a prevalence of 25.3 and 9.3% in 2014, respectively, compared to 23.4 and 7.7% in 2005/06 [37]. On the other hand, chronic conditions are concentrated among older, less educated and poor people, in Portugal [36] and in other countries [38]. Furthermore, there is evidence that old people with chronic diseases are more likely to incur CHE [38], hence, developments in chronic diseases might in part explain the concentration of CHE in households with elderly members and its evolution over time. Evidence also shows that, during the crisis, anxiolytic and antidepressant prescriptions among individuals aged 65 or above doubled between 2011 and 2012 [15]. There is an urgent need to put in place mechanisms to protect these families. The pharmaceutical sector has undergone numerous reforms over the last two decades which were intensified after the financial assistance programme given to Portugal in 2011. Several instruments were adopted, including international reference pricing, changes to retail and wholesale distribution margins, monitoring of prescription patterns, promotion of generic entry and price competition [13]. Despite the price cuts and increase in the share of generics [15], older people apparently remain unprotected against financial hardship. One alternative way might be the adoption of ceilings for annual expenditure on medicines which exist in countries such as Denmark and the Netherlands [38]. Interventions aiming at capacity to pay are most probably not very effective, as income benefits alone have not typically prevented income poverty [39]. Moreover, some of the households with elderly are actually composed of people living alone, which means that they cannot share the risk of catastrophe with other members of their households [32].

In terms of geographical distribution, the most concerning finding is the increase in the risk of CHE in the Centre region of Portugal in 2015 (Azores also had a pronounced increase in 2010/11, but apparently the situation normalised afterwards). It must however be noted that, controlling for other characteristics of households, the AOR for the Centre was not statistically significant, meaning that national measures targeting vulnerable groups based on other risk factors are likely to solve the problem. Still, authorities should be aware of the situation.

Another noticeable change is the growing importance of expenditure on consultations. Given the characteristics of the Portuguese health system (as described before), one would expect the expenditure on consultations to be justified mostly by the use of specialist services. However, the break down of data in 2015 shows a somewhat surprising picture. Why are poor, old people not using the NHS for their GP consultations? Is it a matter of supplier-induced demand like that which has been reported for the case of Greece [30]? This is an issue that remains for future research. Results in Table 3 also show that expenditure on GP visits in the whole sample is regressive. Previous analyses of equity in utilisation of healthcare in Portugal [40–42], consistently identified concentration of GP visits among the poor and concentration of specialist visits among the rich. These results have been interpreted in the light of the Portuguese health system – the poor use more GP services than the rich because these are provided freely or at a reduced cost in the NHS. However, our results suggest that poor people are paying for GP services after all.

Nonetheless, it might be better to pay than go without needed healthcare. In fact, a limitation of the CHE approach is that a low incidence might simply mean that people are not getting the care they need [43]. In Table 2, in all three surveys, a large percentage of households reported zero dental care expenditure. Knowing the shortcomings of the Portuguese NHS in regards to dental care, this is a strong indication of unmet needs. Indeed, in the 2018 Health at Glance Report for Europe [44], Portugal has the highest percentage of unmet needs for dental examination for financial, geographic or waiting time reasons. This means that figures for CHE could be worse if households met their needs, paying for dental care. Another limitation is that, as in other studies [5], we do not include the indirect costs associated with care-seeking like travel costs. In Table 2, we see for example that a very high proportion of households do not spend on hospital services. However, hospitals located outside large metropolitan areas like Lisbon, Oporto and Coimbra do not provide all medical specialties [11] and people living in other regions have to travel to get care in bigger hospitals. Thus, travel and even accommodation costs might be an issue. Moreover, following the financial assistance to Portugal in 2011, patient transportation costs were to be reduced by limiting non-urgent patient transport. The target was achieved and transportation costs decreased by €58 million Euros, between 2010 and 2012 [15]. Another limitation is that we have not looked at persistency of OOP but their duration is important. It has been reported that the greater the time horizon, the greater the effect of risk of catastrophic expenditure [38, 45]. Although we do not have this kind of information, considering our findings about the concentration of CHE among old people and on medicines, there is a high likelihood that these catastrophic payments become long-term expenditure. Households might even suffer a greater welfare loss through the subsequent deterioration of health than those incurring catastrophic payments [25]. Usually, it is assumed that reduced resources owing to OOP has a direct negative impact on household’s welfare. However, it has been noted that medical spending may also increase household health stock and boost productivity mainly in future time periods [46]. The editorial by Peter Zweifel [47] stresses precisely this perspective as he argues that it may make perfect sense to go into debt or accept poverty for a while, provided that health expenditure has a sufficiently strong effect on the recipient’s future labour income. The issue is that when we are talking about older people, as in our study, any future return on OOP is very unlikely to occur.

There are methods, other than the one used in this study, to calculate CHE, depending on how households’ ability to pay is interpreted (OOP are always used as the numerator for calculating the incidence of CHE). The two main approaches are either to consider that the whole budget is available for healthcare spending (budget share approach) or to consider that households must first meet basic needs (capacity to pay approach). In the latter case there might be some further nuances to what is deemed basic needs. Cylus et al. [4] compared the results obtained with the various methods available and concluded that the budget share approach tended to underestimate financial problems among poor people and the opposite among rich people. These authors suggest that capacity to pay approaches should be used especially if the aim is to monitor inequalities within and across countries. In future work, the evolution over time of the incidence and distribution of CHE in Portugal, using several definitions of capacity to pay, might be performed in order to assess whether there are relevant differences according to method. In fact, the discussion about the measurement of CHE is not closed as demonstrated by recent proposals in the literature [48]. However, in this regard there might be a trade-off between more theoretically appealing indicators and simpler yet viable indicators (due to data availability).

Another possible avenue for future research is to explore the association between CHE and its distribution and macro variables such as public debt, economic growth rate, share of public health spending on total health expenditure and percentage of the population benefiting from any insurance scheme besides the NHS.

## Conclusions

This study sought to look beyond the incidence of CHE and to also analyse its distribution in a high income country. Evidence suggests that financial protection, in Portugal, measured through the incidence of CHE, has greatly improved in a decade. At the same time the concentration of CHE among the poorest and households with elderly increased. Consequently, there is the need to reinforce existing protective measures for the elderly poor, and adopt new ones. We also obtained evidence that the weight of spending on medical visits, in particular GP services, is growing. It is of utmost importance go on monitoring this to understand whether the NHS is failing or if individuals, especially elderly, are being induced to consume private services.

Policy makers should pay attention to the distributional dimensions of CHE, as these might provide useful insight to target families at risk. Although inequality is generally regarded as a negative outcome per se, in the context of financial protection, it can actually be regarded as an opportunity for policy making. With greater concentration, interventions to tackle CHE become more confined. It is also important to identify which items are leading families to incur catastrophe health expenditure and monitoring changes over time can contribute to early detection of emerging (and even, unexpected) drivers of catastrophic payments.

## Data Availability

Please contact author for data requests.

## Abbreviations

AOR: Adjusted odds ratios
CCI: Corrected concentration index,
CHE: Catastrophic health expenditure
CI: Concentration index
NHS: National Health Service
OECD: Organisation for Economic Cooperation and Development
OOP: out-of-pocket payments
PHBS: Portuguese Household Budget Survey
WHO: World Health Organisation
